# Spatial transcriptomics herald a new era of transcriptome research

**DOI:** 10.1002/ctm2.1264

**Published:** 2023-05-16

**Authors:** Dominik Saul, Robyn Laura Kosinsky

**Affiliations:** ^1^ Division of Endocrinology Mayo Clinic Rochester Minnesota USA; ^2^ Department of Trauma and Reconstructive Surgery Eberhard Karls University Tübingen BG Trauma Center Tübingen Tübingen Germany; ^3^ Robert Bosch Center for Tumor Diseases Stuttgart Germany

**Keywords:** RNA‐Sequencing, Single Cell Sequencing, Spatial Transcriptomics, Transcriptomics

1

With the beginning of novel RNA‐sequencing in 2006 and 2008,^1^, ^2^ a rapid improvement in transcriptional discoveries led to the development of single‐cell RNA‐sequencing^3^ and single‐nucleus sequencing.^4^ The next ambitious milestone in the omics era was the combination of this high‐throughput sequencing information with spatial data. Understanding the complexities of gene expression will substantially be amended by gaining knowledge of its localization within a tissue or individual cell. In particular, the information will be valuable in the context of a two‐dimensional environment in which the cells of interest naturally occur. One might argue that histological and multiplexed imaging allows the same spatial evaluation of biomarkers; however, the information obtained is limited and biased. While the cellular heterogeneity is traditionally lost in bulk RNA sequencing, and single‐cell RNA sequencing lacks information regarding the tissues’ surroundings or embedding, spatial transcriptomics allows the retrieval of high‐resolution spatially resolved deep RNA sequencing data. In fact, this method does not only enable researchers to identify but also to localize different cell populations within a tissue of interest, which allows conclusions regarding various physiological and pathological processes.

The identification of pathways and whole regulatory networks on a spatially restricted level will provide avenues for drug development as well as the detection of novel biomarkers– and these are just a few of the applications within range. Additionally, the spatial transcriptomic information can be integrated with other modalities such as transposase‐accessible chromatin sequencing (Assay for Transposase‐Accessible Chromatin using sequencing: ATAC‐seq) and protein epitome sequencing (Cellular Indexing of Transcriptomes and Epitomes by Sequencing: CITE‐seq). Moreover, the addition of yet another dimension—time—will be a subsequent cornerstone, currently in sight.^5^

Technically, in order to preserve the architecture of the tissue to be sequenced, RNA needs to be isolated while preserving its spatial information in the tissue. Mostly, oligonucleotide barcodes are used to individually label RNA in the tissue, allowing for the concurrent capture and assessment of RNA levels. Consequentially, the attainable resolution currently limits the applicability and insights of spatial transcriptomics. One of the most commonly used spatial barcoding methods contains a 55 μm spot with 10 000 transcripts but is not able to fully detect all cells. Other spatial barcoding platforms are able to cover 10‐μm spots but with a limited amount of 500 transcripts per bead.^6^ These methodological limitations restrict the researchers’ understanding of cell–cell interactions, tissue composition as well as intracellular spatial heterogeneity. As one solution and opposed to these barcoding‐methods, in situ spatial transcriptomic methods like MERFISH allow the analysis of subcellular transcript patterns at a single cell level, that is, molecular complexes.^7^

While, in general, the advantages of spatial transcriptomics are appealing to the scientific community, there are also caveats that are mostly based on technical restrictions. One disadvantage of spatial barcoding methods (e.g., Visium 10X) is the abovementioned lack of resolution and capture efficiency, which limits the detection of very low expressed transcripts such as *CDKN2A*, a hallmark of senescence and key player in aging biology). In situ spatial transcriptomic methods (e.g., MERFISH and seqFISH+) do not suffer from these limitations of targeting a specific probe set focusing on a particular pathway or genomic pattern.^8^ The downside, however, is optical overpopulation and a lower detection efficiency that is associated with more targeted mRNAs.^8^ There is a limitation of the total amount of RNA that can be recovered, restricting the ability to generate comprehensive whole‐tissue maps.

Due to its novelty and limited technical downstream packages for analysis, an independent validation of the findings from spatial transcriptomics depicts an essentiality to corroborate that the spatial RNA‐distribution accurately reflects the underlying biology. Another considerable limitation is the high cost compared to conventional RNA sequencing methods.

Until rapid sequencing is able to cover long‐lasting cellular processes such as differentiation, devolution and cell cycle progression in real time, trajectory interference serves as an auxiliary to obtain insights into transitions of single cells. Since popular packages like monocle are biased as the user is allowed to choose a starting (or ending) point,^9^ RNA kinetics (using unspliced and spliced RNAs^10^) as used with RNA velocity give a more unbiased representation of a cellular trajectory. Unfortunately, the current sequencing depth occasionally prevents RNA velocity on several available datasets. Nonetheless, to apply these techniques onto spatial datasets will unveil the opportunity to closely “monitor” tissues with a rapid turnover such as the intestinal epithelium (Figure 1).

**FIGURE 1:**
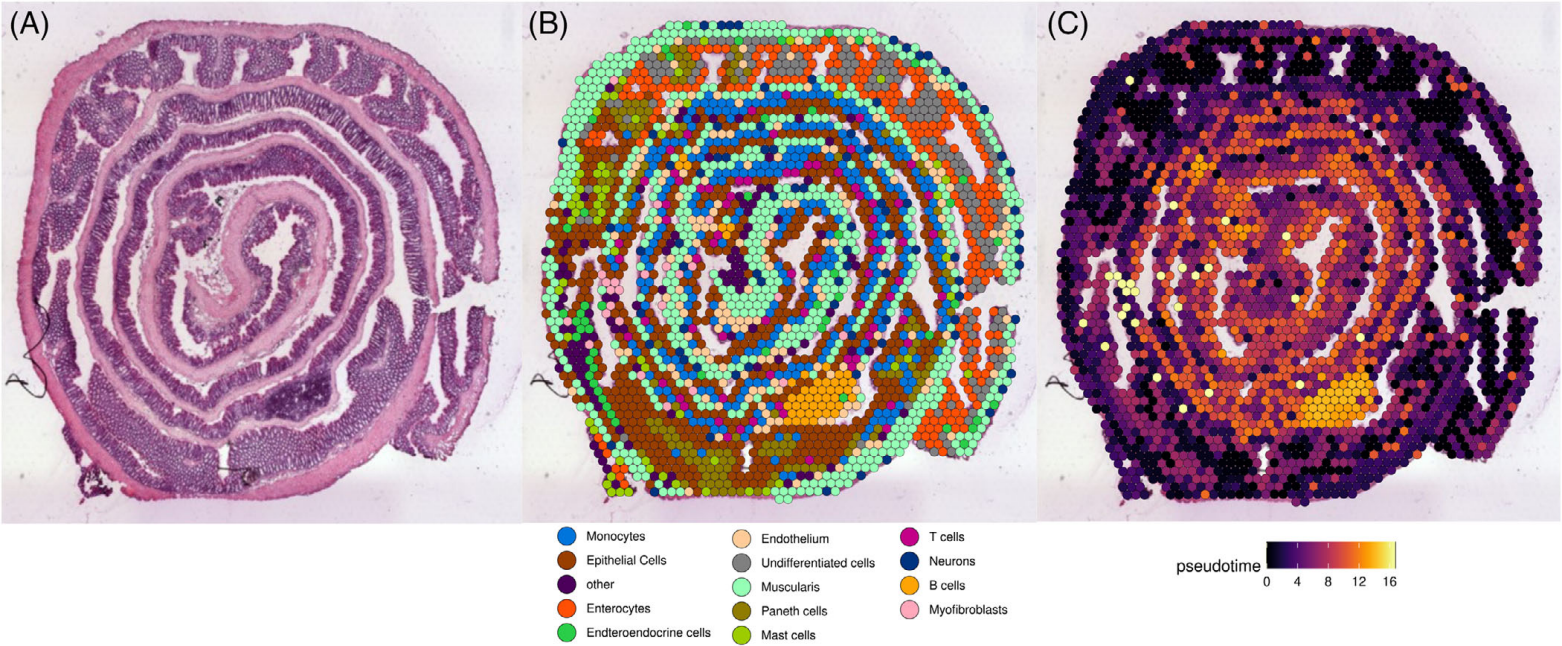
Example of the implementation of pseudotime with spatial transcriptomics. (A) A murine small intestine is prepared as a swiss role and histologically stained via H&E.^14^ (B) Cell type clustering is performed according to Seurat. (C) Trajectory inference via monocle3 is laid on top of the histological section, indicating a late stage for the B‐cell cluster in the inner layer of the swiss role.

The upcoming challenge with the universal availability of larger datasets is to part from a purely descriptive delineation toward a more functional analysis. While cell–cell interaction methods including CellChat and CellPhoneDB paved the way toward a better understanding of small neighborhoods and cellular crosstalk,^11^ tools for unveiling the gene regulatory network underneath the phenotype like SCENIC gained popularity.^12^

With the creation of whole species atlases,^13^ a variety of studies became possible due to the availability and continuously expanding technical opportunities to process these massive datasets. It is only a matter of time until consortia will use cutting‐edge technologies to provide tissue‐specific and potentially age‐specific atlases of not just whole‐transcriptomic but also proteomic, epigenetic, and spatial information of single cells.

In summary, with the increasing availability of spatial transcriptomic datasets, there is substantial potential for unraveling complex biological processes and for the detection and therapeutical exploitation of regulatory genes as their networks. The intricacies of gene expression within a tissue can be simultaneously analyzed, and their spatial localization may provide novel insights into a specific tissue or disease pattern. With several limitations and challenges that need to be addressed, this persistently evolving technique will lead to an expanding and intriguing field of novel applications with a substantial impact on a wide range of scientific fields, from developmental and tissue maintenance to tumor and aging biology.

## References

[1] Bainbridge MN , Warren RL , Hirst M , et al. Analysis of the prostate cancer cell line LNCaP transcriptome using a sequencing‐by‐synthesis approach. BMC Genomics. 2006;7:246. doi:10.1186/1471-2164-7-246 17010196 PMC1592491

[2] Nagalakshmi U , Wang Z , Waern K , et al. The transcriptional landscape of the yeast genome defined by RNA sequencing. Science. 2008;320:1344‐1349. doi:10.1126/science.1158441 18451266 PMC2951732

[3] Haque A , Engel J , Teichmann SA , Lönnberg T . A practical guide to single‐cell RNA‐sequencing for biomedical research and clinical applications. Genome Med. 2017;9:75. doi:10.1186/s13073-017-0467-4 28821273 PMC5561556

[4] Lake BB , Ai R , Kaeser GE , et al. Neuronal subtypes and diversity revealed by single‐nucleus RNA sequencing of the human brain. Science. 2016;352:1586‐1590. doi:10.1126/science.aaf1204 27339989 PMC5038589

[5] Ren J , Zhou H , Zeng H , et al. Spatiotemporally resolved transcriptomics reveals the subcellular RNA kinetic landscape. Nat Methods. 2023. doi:10.1038/s41592-023-01829-8 PMC10172111 37038000

[6] Rao A , Barkley D , França GS , Yanai I . Exploring tissue architecture using spatial transcriptomics. Nature. 2021;596:211‐220. doi:10.1038/s41586-021-03634-9 34381231 PMC8475179

[7] Eng C‐HL , Lawson M , Zhu Q , et al. Transcriptome‐scale super‐resolved imaging in tissues by RNA seqFISH. Nature. 2019;568:235‐239. doi:10.1038/s41586-019-1049-y 30911168 PMC6544023

[8] Kleino I , Frolovaitė P , Suomi T , Elo LL . Computational solutions for spatial transcriptomics. Comput Struct Biotechnol J. 2022;20:4870‐4884. doi:10.1016/j.csbj.2022.08.043 36147664 PMC9464853

[9] Cao J , Spielmann M , Qiu X , et al. The single‐cell transcriptional landscape of mammalian organogenesis. Nature. 2019;566:496‐502. doi:10.1038/s41586-019-0969-x 30787437 PMC6434952

[10] La Manno G , Soldatov R , Zeisel A , et al. RNA velocity of single cells. Nature. 2018;560:494‐498. doi:10.1038/s41586-018-0414-6 30089906 PMC6130801

[11] Liu Z , Sun D , Wang C . Evaluation of cell‐cell interaction methods by integrating single‐cell RNA sequencing data with spatial information. Genome Biol. 2022;23:218. doi:10.1186/s13059-022-02783-y 36253792 PMC9575221

[12] Aibar S , González‐Blas CB , Moerman T , et al. SCENIC: single‐cell regulatory network inference and clustering. Nat Methods. 2017;14:1083‐1086. doi:10.1038/nmeth.4463 28991892 PMC5937676

[13] The Tabula Muris Consortium . Single‐cell transcriptomics of 20 mouse organs creates a Tabula Muris. Nature. 2018;562:367‐372. doi:10.1038/s41586-018-0590-4 30283141 PMC6642641

[14] Parigi SM , Larsson L , Das S , et al. The spatial transcriptomic landscape of the healing mouse intestine following damage. Nat Commun. 2022;13:828. doi:10.1038/s41467-022-28497-0 35149721 PMC8837647

